# The Chemokine CXCL12 Is Essential for the Clearance of the Filaria *Litomosoides sigmodontis* in Resistant Mice

**DOI:** 10.1371/journal.pone.0034971

**Published:** 2012-04-12

**Authors:** Tiffany Bouchery, Gaelle Dénécé, Tarik Attout, Katharina Ehrhardt, Nathaly Lhermitte-Vallarino, Muriel Hachet-Haas, Jean Luc Galzi, Emilie Brotin, Françoise Bachelerie, Laurent Gavotte, Catherine Moulia, Odile Bain, Coralie Martin

**Affiliations:** 1 UMR 7245 MCAM MNHN CNRS & UMR 7205 OSEB MNHN CNRS, Muséum National d'Histoire Naturelle, Paris, France; 2 IREBS, Biotechnologie et Signalisation Cellulaire, UMR 7242, Ecole Supérieure de Biotechnologie de Strasbourg, Illkirch, France; 3 INSERM UMR-S 996, University of Paris-Sud 11, LabEx LERMIT, Clamart, France; 4 UMR 5554 ISEM CNRS, Université Montpellier 2, Montpellier, France; Agency for Science, Technology and Research - Singapore Immunology Network, Singapore

## Abstract

*Litomosoides sigmodontis* is a cause of filarial infection in rodents. Once infective larvae overcome the skin barrier, they enter the lymphatic system and then settle in the pleural cavity, causing soft tissue infection. The outcome of infection depends on the parasite's modulatory ability and also on the immune response of the infected host, which is influenced by its genetic background. The goal of this study was to determine whether host factors such as the chemokine axis CXCL12/CXCR4, which notably participates in the control of immune surveillance, can influence the outcome of the infection. We therefore set up comparative analyses of subcutaneous infection by *L. sigmodontis* in two inbred mouse strains with different outcomes: one susceptible strain (BALB/c) and one resistant strain (C57BL/6). We showed that rapid parasite clearance was associated with a *L. sigmodontis*-specific CXCL12-dependent cell response in C57BL/6 mice. CXCL12 was produced mainly by pleural mesothelial cells during infection. Conversely, the delayed parasite clearance in BALB/c mice was neither associated with an increase in CXCL12 levels nor with cell influx into the pleural cavity. Remarkably, interfering with the CXCL12/CXCR4 axis in both strains of mice delayed filarial development, as evidenced by the postponement of the fourth molting process. Furthermore, the *in vitro* growth of stage 4 filariae was favored by the addition of low amounts of CXCL12. The CXCL12/CXCR4 axis thus appears to have a dual effect on the *L. sigmodontis* life cycle: by acting as a host-cell restriction factor for infection, and as a growth factor for worms.

## Introduction

Filarioids are parasitic Nematodes transmitted by blood-feeding arthropods that deliver infective larvae (L3) into the skin of vertebrate hosts [1]. Some nematode species can host *Wolbachia* bacterial endosymbionts [2], [3]. A common feature of many filarial species is their ability to colonize lymphatic vessels: either they become resident and mature into adults (lymphatic filarioids *e.g. Brugia* spp. and *Wuchereria*), or they use them to reach their privileged niche (such as coelomic cavities) where they complete their development [4]. Although the clinical manifestations can be severe, most of the individuals infected with lymphatic filariasis or onchocerciasis have asymptomatic infections, associated with immune regulatory responses that allow long-term survival of the worms [5]–[9] .

*Litomosoides sigmodontis* is a well-established murine model of filarial infections that mirrors, amongst other things, protective immune mechanisms [6]. Differences in parasite development patterns in resistant (*i.e.* C57BL/6) and susceptible mice (*i.e.* BALB/c) is likely to be inherited dominantly by one gene or closely linked genes as suggested by Choi et al, 2003 [10]. These differences begin early and become progressively more apparent [11]. From day 4 post-inoculation of larvae, surviving L3 begin to appear in the pleural cavity of infected mice. Larvae fully complete their development in BALB/c mice, from infective L3 larvae into L4 larvae, and then into mature, sexually reproducing adult filarial worms. Reduction of filarial load occurs in the pleural cavity, and is much earlier in C57BL/6 mice than in BALB/c mice. Infection is almost resolved in C57BL/6 mice by the time patency starts in BALB/c mice [9]. Another feature of filarial infection in C57BL/6 mice is the higher infiltration of cells in the pleural cavity around the time of the last molt [12]. Cell recruitment is likely to be due to inflammatory stimuli and secretion of chemoattractants such as chemokines.

Chemokines are small proteins that regulate the trafficking of immune cells through interactions with a subset of 7-transmembrane G-protein-coupled receptors [13]. Among them, the CXCL12/SDF-1 chemokine and its receptor CXCR4 are critical players [14]–[16]. CXCL12 is a very potent chemoattractant of neutrophils, monocytes, T-lymphocytes and eosinophils [17]–[21], and mobilization of leukocytes from the bone marrow is largely influenced by interference in the engagement of CXCL12 with CXCR4 [22], [23].

Beyond its role in leukocyte homeostasis, CXCL12 is a pleiotropic chemokine that participates in the regulation of tissue homeostasis (*e.g.* cell survival/proliferation), the importance of which is revealed by its essential role in mouse embryonic development [24]–[27]. CXCL12 is produced in various tissues, which include the bone marrow, the skin and cardiac tissues and the endothelium, peritoneal and pleural mesothelium [28]–[30]. The CXCL12/CXCR4 axis is known to be involved in viral infections, autoimmunity, inflammation, immunodeficiency disorders and cancer. An up-regulation of CXCR4 and CXCL12 was reported in inflammatory diseases, such as rheumatoid arthritis, multiple sclerosis, nephritis and asthma [31]–[33]. Recent studies suggest that disruption of the CXCL12/CXCR4 axis with pharmacological compounds might prove to be an effective treatment strategy for such diseases [32], [34].

In this study, we hypothesized that the CXCL12/CXCR4 axis might be involved in the control of filarial infection. We aimed to define its role using the *L. sigmodontis* infection model of BALB/c and C57BL/6 mice, blocking either CXCL12 with the chelator chalcone C04, or the CXCR4 receptor with the antagonist bicyclam AMD3100.

## Results

### Murine strains differ by their pleural environments and filarial outcomes

**Figure 1:**
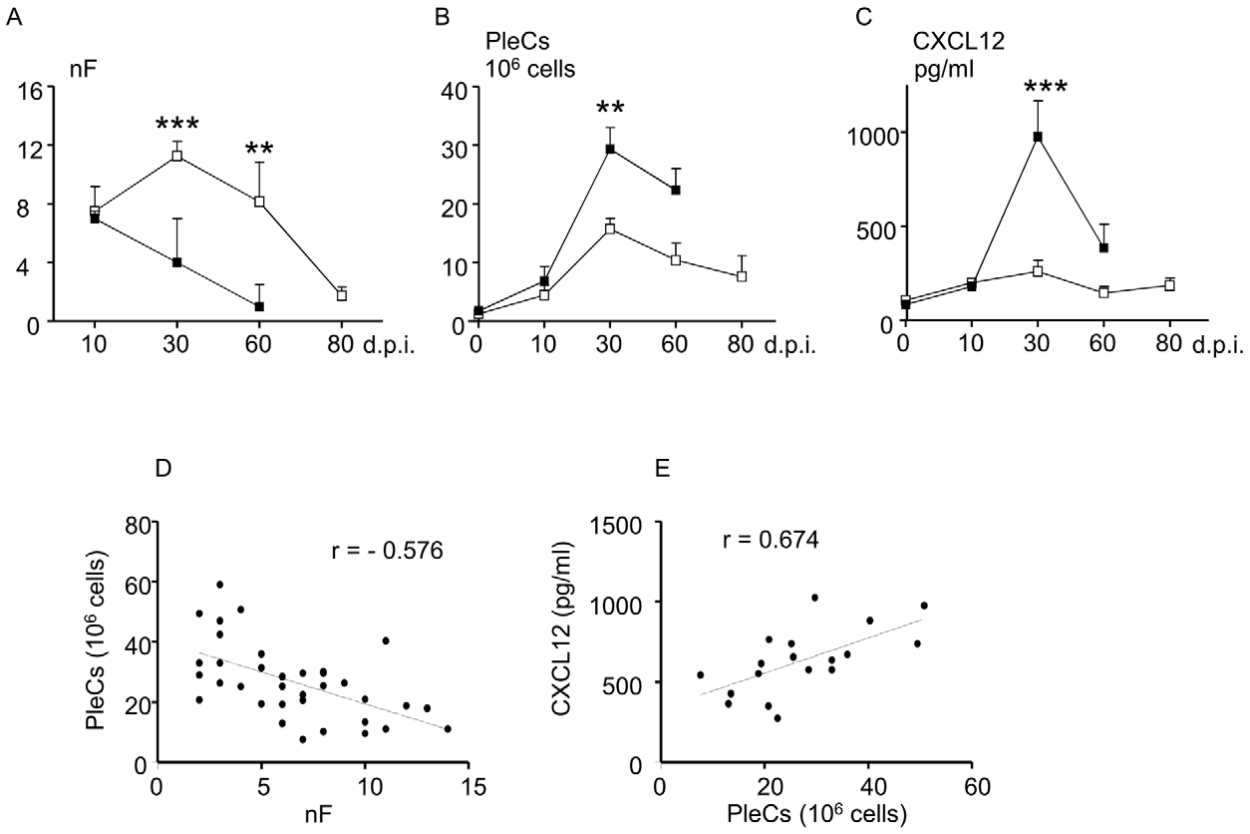
Filarial survival, pleural cell recruitment and CXCL12 levels during the course of the infection. A. Worms were collected during the infection time course from 10 days p.i. to 60 daysp.i in C57BL/6 mice and 80 days p.i. in BALB/c mice and were counted (nF). B. Pleural exudate cells (PleCs) were isolated at necropsy by flushing the pleural cavity and counted. C. The level of CXCL12 was measured during the course of infection in pleural washes of C57BL/6 and BALB/c mice by ELISA (eBiosciences). Open squares represent BALB/c mice, black squares C57BL/6 mice. Results are expressed as mean ± SEM of 3 independent experiments pooled together, each carried out with 6 mice per group. The differences between strains and the modifications during time course of the infection were analyzed by a two-way analysis of variance. For each analysis (nF, PleCs, CXCL12), strain and time effects were significant. Comparison between strains for each time point was further assessed by Bonferroni's multiple comparison test. The character “*” represents significant differences between the C57BL/6 mice and the BALB/c mice (**p<0.005, ***p<0.001). D. Correlation between the filarial load and number of the pleural exudate cells was assessed by Spearman's test (r = −0.576, p<0.01). E. Correlation between the number of pleural exudate cells and the pleural CXCL12 concentration was assessed by Spearman test (r = 0.674, p<0.01). Graphs show the linear regression between the factors.

Larvae were injected subcutaneously in mice and recovered in the pleural cavity 10 days (around molt 3), 30 days (around molt 4), and 60 days (onset of blood microfilariae) post inoculation (p.i.). A later time point (80 days p.i.) was analyzed in BALB/c mice due to the slower clearance of worms in this strain. As described previously [12], the number of recovered worms in BALB/c mice did not vary over the first two months of infection, dropping only between days 60 to 80 (Figure 1A). In contrast, the number of recovered worms in C57BL/6 mice decreased more rapidly and is over before 60 days p.i. (Figure 1A), showing the characteristic faster destruction of worms in this strain of mice.

The total number of cells recovered in the pleural cavity before infection was minimal and identical for each strain (Figure 1B). This number of cells did not vary greatly between 0 h (naive) and 10 days p.i. within strains, or between strains (Figure 1B). As expected [12], cells were recruited in large numbers 30 days p.i. in the pleural space, in both strains of mice, though there was a significant strain effect (Figure 1B). Indeed, at that time point, recruitment was higher in C57BL/6 than in BALB/c mice (Figure 1B, 29.3±14 *vs* 15.7±6.4×10^6^ cells/mouse). At 30 days post-inoculation, the number of pleural exudate cells correlates negatively with the number of filariae recovered in the pleural cavity (Figure 1D, r = −0.576, p<0.01). The total number of cells recovered from the pleural cavity at 60 days p.i. decreased in both strains of mice (Figure 1B).

In a previous study, we characterized the infiltrated cells at 10 and 30 days p.i. and found that the kinetics of T and B cell recruitment are different between the two strains: slow then fast in C57BL/6 mice *vs* fast then slow in BALB/c mice [12]. However, at 30 days p.i., no difference in the proportion of each cell type was observed between the two strains of mice [12]. The higher peak of cell influx in the pleural cavity of C57BL/6 mice was associated with a higher secretion of inflammatory chemokines CCL2, CCL3 and CCL11 (Figure S1 and [12]) in the pleural fluid.

The chemokine CXCL12 attracts a large number of immune cells and has been shown to be involved in the recruitment of leukocytes in lung and peritoneal inflammation [35]. CXCL12 is highly increased in the pleural fluid of C57BL/6 mice 30 days p.i., while the level remains unchanged in BALB/c mice, even at day 80 p.i. when the filarial load has fallen (Figure 1C). At 30 days post-inoculation, the level of CXCL12 in the pleural cavity correlates with the number of pleural exudate cells (Figure 1E, r = 0.674, p<0.01).

### Disruption of the CXCL12/CXCR4 pathway favors worm survival in C57BL/6 mice

**Figure 2:**
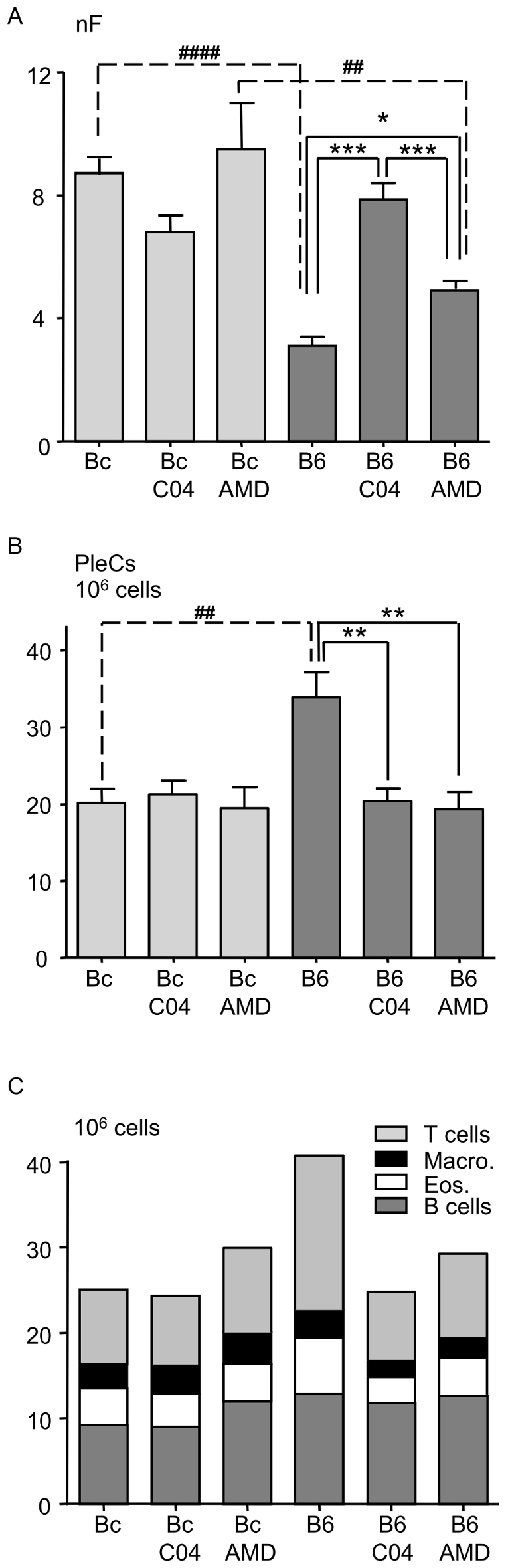
Reversal of resistance upon CXCL12/CXCR4 axis disruption. A. Filarial load 30 days post-inoculation in BALB/c mice (Bc, light gray) compared to C57BL/6 mice (B6, dark grey) after the disruption of the CXCL12/CXCR4 axis. Mice were treated with 100 µg AMD3100 (AMD) or 2.5 mg chalcone C04 (C04) at 10 and 20 days post-inoculation as described in Material and Methods. Results are expressed as a barplot of 3 pooled independent experiments each carried out with 6 mice per group. The differences between strains and those between treatments were analyzed by a two-way analysis of variance. Strain and time effects were significant. Further comparisons were assessed by Bonferroni's multiple comparison test. The character “*” represents significant differences between treatments within one strain of mice (*p<0.05, **p<0.005, ***p<0.001); “#” shows significant differences between strains for one given treatment (##p<0.005, ###p<0.001). B. Pleural exudates cell (PleC) count. PleCs were counted at necropsy 30 days post-inoculation in BALB/c mice (Bc, light grey) and in C57BL/6 mice (B6, dark grey) after the disruption of the CXCL12/CXCR4 axis by AMD3100 (AMD) or chalcone C04 (C04). Results are expressed as a barplot of 3 pooled independent experiments, each carried with 6 mice per group. The differences between strains and those between treatments were analyzed by a two-way analysis of variance. Strain and time effects were significant. Further comparisons were assessed by Bonferroni's multiple comparison test. The character “*” represents significant differences between treatments within one strain of mice (**p<0.005); “#” shows significant differences between strains for one given treatment (##p<0.005). C. Pleural exudates cell composition. PleCs were characterized by FACS analysis. Cells were labelled with various antibodies and then analysed by flow cytometry (FACSCanto BD, FACS DIVA version 6.0) as described in Material and Methods and Figure S2. B cells are defined as cells expressing CD19, T cells as expressing CD3, macrophages as expressing F4/80, eosinophils as expressing Siglec F and neutrophils as expressing Ly6G. Although *L. sigmodontis* infection does not mobilize neutrophils in the pleural cavity at 30 days p.i., these granulocytes were analyzed to determine if treatments can induce their recruitment. Due to their very low numbers in all groups, neutrophils were not represented on the graph. Results are expressed as the mean of 6 observations for each cell type and represented as a stacked barplot. A multiple analysis of variance (MANOVA) was used to determine if the proportion of each pleural exudate cell type is modified between murine strains or by the treatments: the proportion of the different PleC types was not modified by the treatments, neither in Bc nor in B6 mice.

We thus investigated whether the secretion of CXCL12 in the pleural cavity of C57BL/6 mice might have a hand in the fate of *L. sigmodontis* infection by assessing the consequences of a blockade of the CXCL12/CXCR4 axis in both BALB/c and C57BL/6 mice. This was done using inhibitors that interact either with the chemokine (*i.e.* chalcone C04 [36]) or the receptor (*i.e.* AMD3100). We found that intraperitoneal treatments with either chalcone C04 (at 10 and 20 days p.i., with a2.5 mg/mouse dose) or AMD3100 (at 10 and 20 days p.i., with a 100 µg/mouse dose) both significantly increased worm load in C57BL/6 mice. Upon chalcone C04 administration, the worm load in C57BL/6 mice reached the one observed in BALB/c mice (Figure 2A). The AMD3100 treatment appeared to be less potent than the chalcone C04 one. However, this is likely the consequence of the different half-lives of the products and their bioavailability, together with intrinsic differences in their mechanisms of interference on filarial load. Neither of the treatments modified the filarial load in BALB/c mice. These results support the hypothesis that CXCL12 has a role in the resistant phenotype of the C57BL/6 mice.

The treatments with chalcone C04 and AMD3100 reduced the total number of pleural exudate cell (PleCs) collected in the pleural lavage fluid of the C57BL/6 mice (Figure 2B). However, PleC composition was not modified by the treatments (Figure 2C, Figure S2).

### Pleural mesothelial cells are CXCL12 providers in C57BL/6 mice

**Figure 3:**
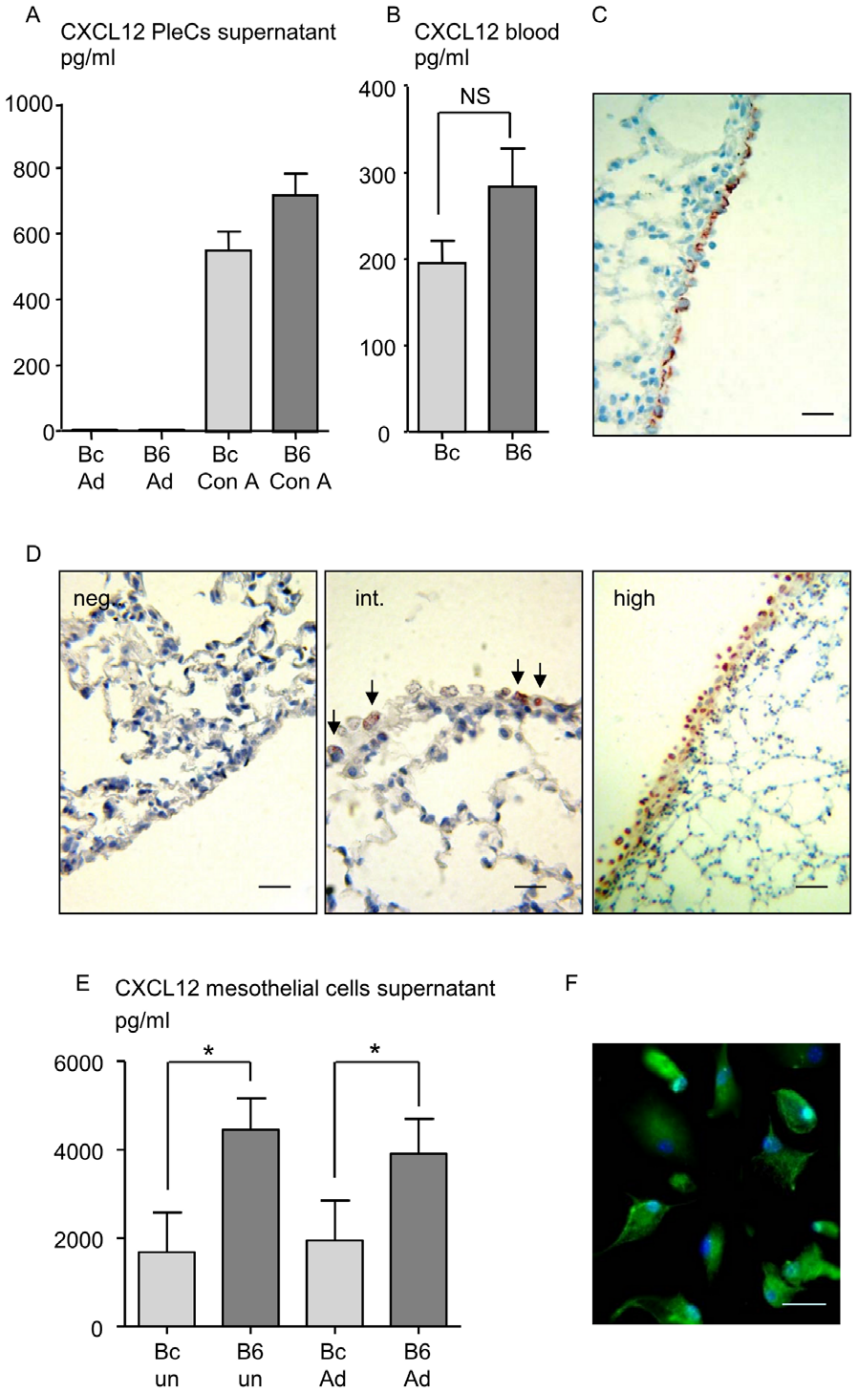
CXCL12 is produced by the mesothelium of C57BL/6 mice. A. Pleural exudate cells (PleCs) were harvested 30 days post-filarial inoculation (p.i.), by PBS washing. The cells were stimulated for 72 hours with crude extract of adult (Ad) *L. sigmodontis* (10 µg/ml) or with 1 µg/ml of the mitogen Concanavalin A (Con A). Levels of CXCL12 were detected by ELISA (eBiosciences) in the culture supernatant. Results are expressed as mean ± SEM, 6 mice per group. B. Differential levels of CXCL12 at 30 days p.i. in the sera of BALB/c mice (Bc) and C57BL/6 mice (B6) measured by ELISA. Results are expressed as mean ± SEM, 6 mice per group; unpaired t-test, non significant (NS). C. Chromogenic immunohistochemical staining with cytokeratine 7 of paraffine-embedded visceral pleural mesothelium sections representative of naive or infected BALB/c and naive C57BL/6 tissues. Scale: bars = 20 µm. D. Chromogenic immunohistochemical staining of paraffine-embedded tissue section of C57BL/6 and BALB/c visceral pleural mesothelium: figures are representative of established scores as described in Material and Methods and in Table 1. From left to right: negative (neg.) CXCL12 staining, weak CXCL12 staining (int. for intermediate), high CXCL12 staining (high). Same results were observed with the two antibodies tested (polyclonal antibody, 1∶500, eBioscience; biotinylated clone K15C, 1∶200). Scale: bars = 20, 10, 40 µm. E. Differential levels of CXCL12 in mesothelial cell supernatants was measured by ELISA. Mesothelial pleural cells were harvested by trypsine digestion from 12 BALB/c mice and 12 C57BL/6 mice 30 days post-filarial inoculation. Once at 70% of confluence, half of the cells were stimulated for 48 hours with crude extract of adult *L. sigmodontis* (10 µg/ml, groups Bc Ad and B6 Ad). Unstimulated cells were also tested (Bc un and B6 un). Detail of culture conditions is provided in Material and Methods. Results are presented after subtraction of the baseline production as a barplot showing medians and range. The differences between strains were analysed by a Kruskall-Wallis test followed by a Dunn's multiple comparison test. “*” represents significant differences between the C57BL/6 mice and the BALB/c mice. F. Fluorescent immunological staining of CXCR4 on mesothelial cells culture. Scale: bars = 15 µm.

While CXCL12 levels are highly increased in the pleural fluid of C57BL/6 mice 30 days p.i (Figure 1C), neither the release of CXCL12 by infiltrated pleural cells (Figure 3A, Figure S3) nor blood levels (Figure 3B) can explain such concentrations. It is known that one main source of CXCL12 are mesothelial cells [28]. Normal mesothelial cells are present as a single layer applied to a thin band of fibrous tissue and are characterized by cytokeratin 7 expression (Figure 3C). Although expression of CXCL12 was observed in visceral pleural mesothelium from both naive BALB/c and C57BL/6 mice (Table 1), it ranged from undetectable to low levels whatever the mice strain (Figure 3D left and middle). Infection outcome 30 days p.i. did not modify CXCL12 expression in BALB/c mice (Table 1), whereas C57BL/6 mice displayed more CXCL12 in their mesothelium (Table 1, Figure 3D right). In addition, slight hyperplasia of visceral pleural mesothelium and nuclear pleomorphism were observed in infected C57BL/6 mice (Figure 3D right). Once cultured, the pleural mesothelial cells from infected-C57BL/6 mice released more CXCL12 than the cells from infected BALB/c mice, whether they were re-stimulated by *L. sigmodontis* crude extract or not (Figure 3E). In addition, we checked that cultured mesothelial cells express CXCR4 in both BALB/c and C57BL/6 mice (Figure 3F).

**Table 1:** CXCL12 intensity score in mesothelial cells.

	Number of sections of lobes				
Mice	Total	Neg. (score 0)	Int. (score 5)	High (score 10)	Mean score/lobe
naive BALB/c	10	5	5	0	3
naive C57BL/6	11	9	2	0	1
inf. BALB/c	17	10	7	0	2
inf. C57BL/6	15	0	7	8	8
inf. C57BL/6 C04	8	6	2	0	1
inf. C57BL/6 AMD	8	3	5	0	3

The visceral pleural mesothelium from infected (inf.) and naive C57BL/6 and BALB/c mice was stained for CXCL12 (polyclonal antibody, 1∶500, eBioscience; and biotinylated monoclonal 1∶200, clone K15C). Four animals were studied for each condition (6 groups: naive BALB/c, naive C57BL/6, infected BALB/c, infected C57BL/6, infected and C04 or AMD3100-treated BALB/c and C57BL/6). 8 to 17 whole sections of pulmonary lobes were analyzed by light microscopy and discriminated according to the intensity of CXCL12 staining in 3 categories: no detectable staining (Neg.), intermediate (Int.) or high intensity (High). The level of CXCL12 was arbitrarily scored as 0 (no detectable staining), 5 (intermediate staining), and 10 (high staining). A mean score (weighted mean) per section of pulmonary lobe was calculated according to the coefficient defined previously.

### C04 and AMD3100 treatments prevent CXCL12 but not IL-5 pleural production

**Figure 4:**
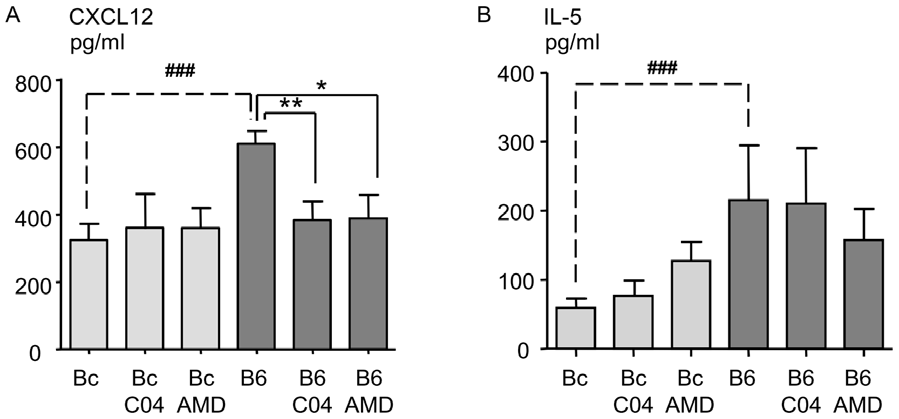
Pleural levels of CXCL12 and IL-5 after disruption of the CXCL12/CXCR4 axis. CXCL12 (A) and IL-5 (B) expression levels measured by ELISA in pleural fluid of BALB/c mice and C57BL/6 mice 30 days post-inoculation and upon blockade either by chalcone (C04) or by AMD3100 (AMD) (days 10 and 20 p.i.). Results are presented after subtraction of the baseline production as mean ± SEM of 3 pooled independent experiments each carried out with 6 mice per group. The differences between strains and treatments were analysed by a two-way analysis of variance. Strain and treatment effects were significant for the pleural CXCL12 levels, and only the strain effect was significant for the pleural IL-5 level. Further comparisons were assessed by Bonferroni's multiple comparison test. “*” represents significant differences between treatments within one strain of mice (*p<0.05, **p<0.01); # shows significant differences between strains for one given treatment (###p<0.001).

Treatment of infected C57BL/6 mice with either C04 or AMD3100 had an impact on the intracellular levels of CXCL12 in the pulmonary mesothelium, which shrunk to those observed in naïve mice (Table 1). Accordingly, CXCL12 levels in the pleural wash of infected C57BL/6 mice was also reduced by the two treatments (Figure 4A). Because IL-5 has previously been shown to be involved in the resolution of *L. sigmodontis* infection [37]–[39], the presence of this cytokine was analyzed in the pleural wash. Although IL-5 levels were significantly higher in C57BL/6 mice than in BALB/c mice, the two treatments did not modify the secretion of this cytokine (Figure 4B).

### Disruption of CXCL12/CXCR4 axis delays worm development in both strains of mice

**Figure 5:**
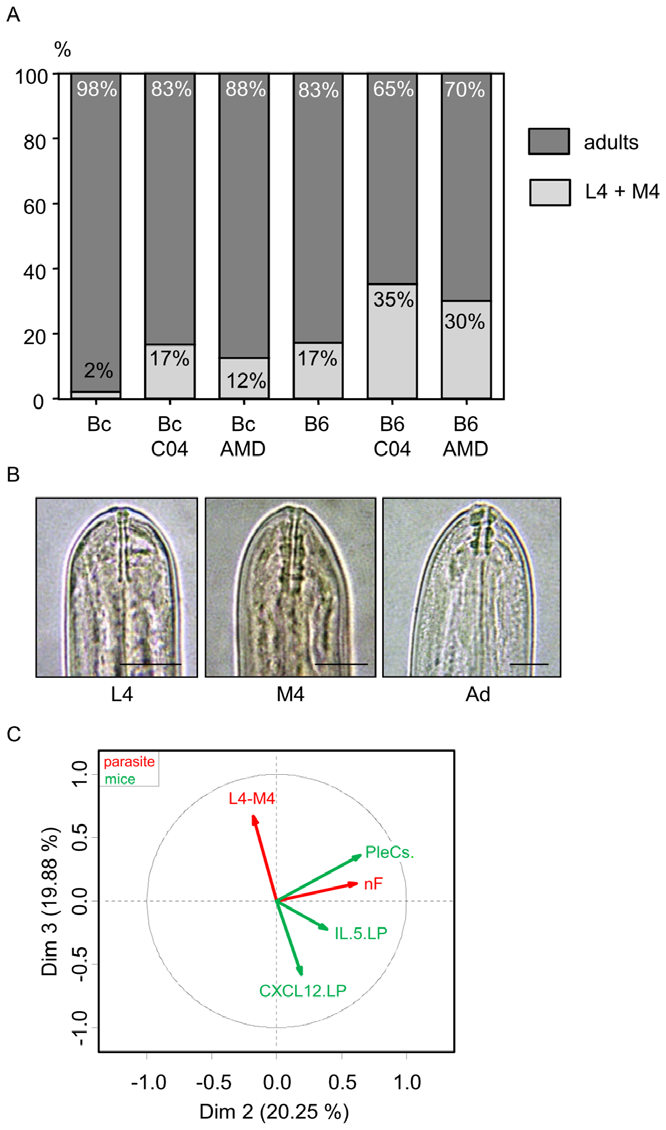
The CXCL12/CXCR4 axis affects filarial development. A. Percentage of adults (dark grey) and non adults (light grey), *i.e.* stage 4 (L4) or molt 4 (M4), recovered 30 days post-inoculation after treatment with chalcone (C04) or AMD3100 (AMD). Results are presented in stacked column chart; binomial glm (see Materials and method for details). B. Morphology of *L. sigmodontis* buccal capsule of fourth stage (L4), during fourth molting (M4), and of adult (Ad) by light microscopy. The fourth stage has a thin buccal capsule, composed of two thin walls (dark arrow), the fourth molt simultaneously presents the buccal capsule of the fourth stage (dark arrow) and the buccal capsule characteristic of the adult stage with three large segments. Scale: bars = 20 µm. C. Multiple factorial analysis (MFA) of the parameters implicated in the study: pleural CXCL12, pleural IL-5, number of pleural exudate cells (PleCs), number of filariae (nF), and the percentage of stage 4 and molt 4 (L4–M4). For a non-biased representation of the data observed either on mice or on filariae, PleCs, IL-5, and CXCL12 factors were grouped together on one hand, and the number of filariae and L4–M4 factors on the other hand. Controlled variables, such as strain or treatment, were considered as illustrative.

*L. sigmodontis* larval stage 4 is expected to molt into the adult stage around day 28 post inoculation in BALB/c mice; however, it is also known that the timing of this molt is dependent on the genetic background of the host [40]. Indeed, 17% of worms from C57BL/6 mice were not yet adults 30 days p.i. whereas almost all worms recovered from BALB/c mice were adults (98%) ([12] and Figure 5A). The developmental stage is determined based on the morphology of the buccal capsule, which appears as two thin lines in stage 4, and consists of 3 identifiable segments in adults. At molt 4, larvae exhibit buccal capsules of both types (Figure 5B). Surprisingly, in both strains of mice treated either by the chalcone C04 or by AMD3100, the percentage of larval stage 4 and fourth molting filariae increased 30 days p.i. (Figure 5A), from 17% to 35% and 30% in worms recovered from C57BL/6 mice and from 2% to 17% and 12% in worms recovered from BALB/c mice, respectively treated with chalcone C04 or AMD3100. This suggests that CXCL12 can be sensed by filariae as a cue for development.

In order to understand the links between growth delay and the treatment, a multiple factorial analysis was performed. The first dimension constitutes an axis of resistance (29.92% of total variance), from the susceptible BALB/c mice on the left with high filariae counts (nF), to resistant C57BL/6 mice on the right characterized by high levels of CXCL12 and IL-5 (Figure S4). To discriminate the effect of the disruption of the CXCL12/CXCR4 axis (treatment), it was thus necessary to study axes 2 (20.25% of total variance) and 3 (19.88% of total variance). CXCL12 levels and the proportion of L4–M4 at 30 days p.i. are inversely correlated and captured by the third axis. To a less significant degree, IL-5 is also inversely correlated to the proportion of L4–M4. Altogether, these results suggest a role of CXCL12, and to a lesser extent of IL-5, in the development of filariae.

### Low concentrations of CXCL12, but not high ones, improve *in vitro* worm growth

**Figure 6:**
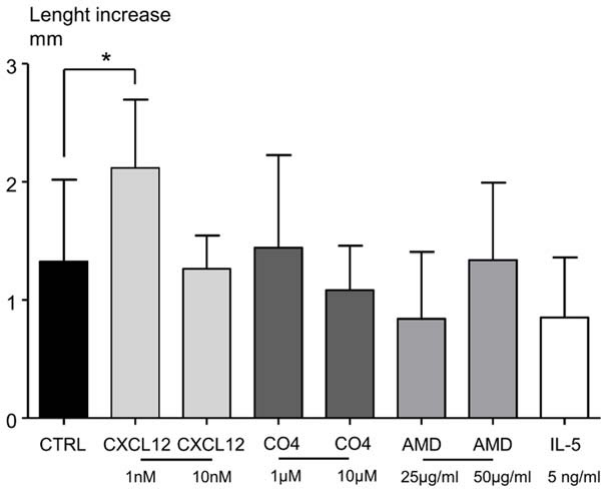
CXCL12 has direct effect on worm growth *in vitro*. Length of filariae (mm) was measured before and after 5 days of *in vitro* culture. Groups of filariae were constituted and treated with either CXCL12 (1 nM, 10 nM), Chalcone C04 (C04, 1 µM, 10 µM), AMD3100 (AMD, 25, 50 µg/ml) or IL-5 (5 ng/ml). Treatments were added daily to wells containing two filariae in 1 ml, as detailed in Materials and Method. Length increase was expressed as median ± interquartile range of 30 filariae for control group (Ctrl, medium only), 20 for other groups, and 10 for the IL-5 treated group. The differences between treatments were analysed by a non parametric Kruskall-wallis test followed by Dunn's multiple comparison test, with control group as reference. “*” marks significant differences between treated filariae and the control group (*p<0.05).

L4-stage larvae (of length 3.8 mm±0.07) were cultured for five days either with CXCL12 (1 or 10 nM), AMD3100 (25 µg/ml), Chalcone C04 (1 or 10 µM) or IL-5 (5 ng/ml). The medium used for culturing the worms was tested by ELISA for the presence of CXCL12 and was shown to be negative. The length of untreated worms had increased by 1.2 mm at the end of the culture (Figure 6). Low concentrations of CXCL12 significantly improved the growth, increasing it to 2.12 mm, whereas high concentrations did not, underlining a different effect of CXCL12 on filarial growth according to its concentration (Figure 6). Chalcone C04, AMD3100 and recombinant IL-5 had no impact on worm growth (Figure 6). All filariae from these experiments presented rapid and regular movements, and were thus neither dead nor moribund.

## Discussion

This study highlights a key role of CXCL12 in the control of filarial infection. Indeed, CXCL12 proved to be a host cell factor which is critical for a fast clearance of the parasite in C57BL/6 mice (Figure S5A). In addition, a dual role for the CXCL12/CXCR4 axis is suggested, as it modulates the growth of the parasite in a mouse strain-independent manner, and thus is more likely to have a direct effect on the parasite (Figure S5B).

Several studies have shown that the CXCL12/CXCR4 axis is involved in the progression of diseases and infections. In murine models of asthma, disruption of this axis has been shown to decrease lung damage and airway reactivity, as illustrated by reduction of airway resistance, decreased microvessel density and general alteration in the development of airway inflammation [36], [41], [42]. In murine models of malaria, the supplementation of lethal infection models with CXCL12 induces a clear reduction in parasitemia. Conversely, blocking CXCL12 action by administration of a CXCR4 receptor blocker causes an increase in circulating parasites in the usually benign non-lethal *P. chabaudi* malaria in C57BL/6 mice [43]. Similarly, in the *L. sigmodontis* model, blocking the action of CXCL12 by either administrating the C04 chalcone or a CXCR4 antagonist induces an increase in the filarial load in resistant CXCL12-responsive C57BL/6 mice (Figure 2A, Figure S5A). Thus, modification of the CXCL12/CXCR4 axis grants control over the pathology's development in these infection models. The importance of CXCL12/CXCR4 axis in filarial pathology is further supported by the fact that chronically infected (lymphatic filariasis) but nevertheless asymptomatic individuals have a lower expression of CXCR4 on T cells as compared to both uninfected and resistant individuals [44].

The *L. sigmodontis* filarial infection is a Th2-based helminthiasis [6] and the CXCL12/CXCR4 axis has been reported to have a role in numerous Th2-based inflammatory diseases [32], [42]. In particular, disruption of the axis causes a decrease in cell recruitment, as seen in asthma [41], [42] or antigen-elicited schistosomal granuloma formation (AESGF) [45]. Similarly, a reduction of pleural cell recruitment was demonstrated herein in *L. sigmodontis* filarial infection in treated resistant mice (Figure 2B). Eosinophils are key players of a protective response against the infective and adult stages of *L. sigmodontis* [37]–[39]. However, the eosinophil population was not preponderantly impacted by the blockade of the CXCL12/CXCR4 axis (Figure 2C), unlike in asthma and AESGF [36], [42], [45]. Nevertheless, as with AESGF [45], no clear deficit in Th2 cytokines was observed in the pleural compartment, as indicated by unmodified IL-5 levels (Figure 4). This difference may be related to the type of diseases studied, since asthma and AESGF are inflammatory responses mainly caused by eosinophils [36], [42]. Furthermore, the kinetics of cell recruitment studied in these diseases are short, up to 24 hours post-challenge [36], [42]. Besides eosinophils, the various cell types recovered in the pleural cavity after disruption of the CXCL12/CXCR4 axis were all reduced in the same proportion (Figure 2C and Figure S2).

Several species of filarioids establish themselves in lymphatics or in coelomic cavities, which are bordered by endothelial or mesothelial cells. These cells share numerous properties like the capacity to produce a broad spectrum of chemokines and chemokine receptors under inflammatory conditions, such as CXCL8, CCL3, CXL12 for the mesothelial cells [28], [46], CXCL1, CXCL10, CXCL14 for endothelial cells, and CCL2 or CCL5 for both types [47], [48]. Mesothelial cells are distributed in a monolayer that lines the pleural cavity. It allows dilatation/contraction of the lung during breathing, regulates pleural permeability and provides protection against pathogens [46]. For example, secretion of chemokines by mesothelial cells has been shown to promote leukocyte influx from the vascular compartment to the pleural cavity via transmesothelial migration after infections such as tuberculosis or bacterial peritonitis [49], [50]. *L. sigmodontis* migrates from the skin through the lymphatic system to the pleural cavity where it matures and induces inflammation. Inflammatory response is selectively stimulated, in respect to the strain of mice. Indeed, the faster clearance of filarial infection appears in C57BL/6 mice, within which a strong increase of chemokine concentrations, including CXCL12, is measured in the pleural cavity, peaking at the time of the 4th molt, *i.e.* around one month post infection. Inflammatory chemokines such as CCL11 have been detected in pleural fluid [12] and can be produced by pleural exudate cells (Figure S1), whereas CXCL12 is produced by mesothelial cells when infected by *L. sigmondontis* (Figure 3). In contrast, the clearance of *L. sigmodontis* is comparatively slow in susceptible BALB/c mice, which display a moderate increase of chemokines throughout the infection (Figure 1, Figure S1). One could thus hypothesize that increasing pleural CXCL12 levels in susceptible BALB/c might confer them increased resistance against the filaria. However, this approach is technically compromised by the short half-life of recombinant CXCL12. We don't know yet the underlying mechanisms explaining the lack of CXCL12/CXCR4-mediated immune response in BALB/c mice, but for example the absence of a functional copy of Cxcl11 in C57BL/6 mice, contrary to BALB/c mice could be an explanation. Indeed, as this chemokine signals through CXCR7, the second receptor of CXCL12 [51], this difference may affect the state of activation of the CXCL12/CXCR4 axis. The importance of the CXCL12/CXCR4 axis in the susceptibility/resistance phenotype against filariasis is further supported by the recent observation of a correlation between high CXCR4 expression in PleCs and a low filarial recovery rate in granzyme-deficient C57BL/6 mice presenting different degrees of susceptibility after infection by *L. sigmodontis* [52].

This work is the first to highlight that mesothelial cells can act as an important source of chemokines during filarial infection, and suggests that the clearance of filariae is dependent on the capacity of these cells to respond to the filariae. Mesothelial cells can produce chemokines following activation of Toll-like receptors (TLR) such as TLR2/1 and TLR2/6 [53], [54]. The endobacteria *Wolbachia* is able to signal through TLR2 and TLR6 [55] opening the possibility that it contributes to the mesothelial responses. Further work is needed to assess whether damaged filariae, and the consequent exposure of *Wolbachia*, participates in the mechanism that controls host-dependent chemokine production during the course of the infection.

In addition to its role in filarial clearance, the CXCL12/CXCR4 axis impacts the growth of the parasite independently of the mouse strain, likely through a direct effect on the filariae (Figure S5B). Filarioids can increase in size between molts [56]. However, the end of the molting process can be more or less precocious depending on the host background. For example, *L. sigmodontis* 4^th^ molt happens faster in rodents in which filarial infection is cleared slowly [40], [57]. Conversely, in resistant C57BL/6 mice, a molting delay has been reported in previous studies [12] and in the current one (Figure 5A).

The use of the *L. sigmodontis* murine infection model suggests that immune parameters can be environmental factors influencing the development and the fitness of the parasite. IL-5 has been shown to control the growth of *L. sigmodontis* only in presence of eosinophils [58]. IL-4 and IL-5 are also important in the regulation of worm fertility, because infected IL-4 KO and IL-5 KO mice produce more microfilariae, and for a longer period of time [59]. In the current study, CXCL12 was shown to be able to modify the growth in a non-linear dose-dependent manner within a controlled *in vitro* environment (Figure 6). Indeed, as *in vivo*, a high concentration of CXCL12 (*i.e.* in C57BL/6 mice) limited the growth of the parasite, while a low concentration of CXCL12 (*i.e*. in BALB/c mice) favored it. Furthermore, neither C04 chalcone nor AMD3100 had a direct effect *in vitro* when incubated with filariae in the absence of CXCL12. This suggests that filariae possess a CXCR4-like receptor able to sense pleural CXCL12 (Figure S5B). At high concentrations of this chemokine the consequences on filarial growth are negative, most probably as a result of receptor desensitization. The waning of responses from cell surface activated receptors during persistent stimulation with agonists (e.g. desensitization) is a feature shared by many G protein coupled receptors, including the chemokine receptor family. For instance, impaired desensitization of CXCR4 leads to abnormally enhanced responses to CXCL12 [60]–[62]. Thus, under our hypothesis, preventing the signalling of CXCL12 on its receptor by using AMD3100 or the chalcone C04 would also impair worm development, whatever the mouse strain, as is indeed observed.

The presence of an ortholog/mimic of CXCR4 in the filarial genome was assessed on *Brugia malayi* genome, as the genome of *Litomosoides sigmodontis* is not yet available. However, genome-wide BLAST searches conducted on the Nembase sequence databases (www.nematodes.org/nembase4/) failed to yield a significant match for human or murine CXCR4 sequences (data not shown). Nevertheless, CXCR4 is the most conserved chemokine receptor among vertebrates and is even known to be present in ancestral fish families, such as the chondrostian and elasmobranch taxa, which diverge early in vertebrate evolution [63], [64]. Invertebrates are generally considered to be free of G-protein-coupled receptors (GPCRs) of the rhodopsin γ family, which includes chemokine receptors [65]. However, the urochordate sea squirt *Ciona intestinalis*, one of the closest invertebrate relatives to vertebrates, has been reported to have GPCRs from this family [66], thus stressing the possibility that invertebrates might possess chemokine-like pathways. Furthermore, even if orthologs/mimics of mammalian chemokine receptors have not been identified in Nematodes yet, chemokine-like proteins and receptors of Nematodes are known to be able to interfere with mammalian chemokine pathways, raising the possibility that filarioidea possess a CXCR4 mimic able to interact with mammalian system.

In conclusion, this study demonstrated that the abrogation of the CXCL12/CXCR4 axis in *L. sigmodontis* infection of fast-clearing mice reverses the resistant phenotype, with lower pleural cell recruitment and higher worm survival rate. Conversely, this increased survival is compromised by an alteration of worm development, independently of the host's genetic background. As the differences of susceptibility in the *L. sigmodontis* mouse model can reflect the large panel of clinical manifestations [6]–[9], the study of CXCL12-dependant mechanisms of filarial destruction in resistant mice might yield interesting new therapeutic targets.

## Materials and Methods

### Ethics Statement

All experimental procedures were carried out in strict accordance with the 2003/65/CEE European directive for animal experimentation. National license number 75–1415 approved animal experiments. Protocols were approved by the ethical committee of the “Museum National d'Histoire Naturelle” (MNHN) and by the “Direction départementale de la cohésion sociale et de la protection des populations” (DDCSPP) (n°75-05-15).

### Parasites, mice, infection, treatments

*Litomosoides sigmodontis* was maintained in our laboratory, and infective third-stage larvae (L3) were recovered by dissection of the mite vector *Ornithonyssus bacoti* as previously described [38], [67].

Crude extract of *L. sigmodontis* worms were obtained by homogenization and sonication of adults recovered from jirds (sex ratio 1∶1) as previously described [12]. After centrifugation, the supernatant was collected and frozen at −80°C until further use. Protein content was determined by the modified Bradford method (BCA™ Protein Assay kit, Pierce).

Six-week-old female C57BL/6 and BALB/c mice were purchased from Harlan (France) and maintained in the MNHN animal facilities. Forty infective L3 in 200 µl of RPMI 1640 were inoculated subcutaneously into the left lumbar area of mice. Kinetics of infection were followed over 80 days of infection. Mice were sacrificed at 10, 30, 60, and 80 days post-inoculation (p.i.).

Groups of mice were twice treated intraperitoneally with 2.5 mg of chalcone C04 dissolved in 1% Carboxy Methyl Cellulose (Sigma) 10 and 20 days p.i. according to the protocol described by Hachet-Haas *et al*. [36] or with 100 µg of AMD3100 in 0.9% NaCl (Sigma) using the same schedule. Mice were sacrificed 30 days p.i.

At least six mice were used for each group, and each experiment was repeated 3 times.

### Filarial load, pleural leukocyte recovery, flow cytometry

The mice were anesthetized and sacrificed by final bleeding. The pleural cavity was washed with 10 ml of cold phosphate buffered saline (PBS), as previously described [38], [68]. The infiltrating cells as well as the worms were collected from the pleural wash for further analysis.

The following parasite features were analyzed by light microscopy on materials fixed *in toto* with 4% formaldehyde in cold PBS to avoid body shrinkage: i) L4/molt 4/adults; ii) gender. Pleural exudates cells (PleCs) were centrifuged at 250 g for 8 min at 4°C, resuspended in 1 ml RPMI supplemented with 10% foetal calf serum (FCS) and counted in PBS−0.04% trypan blue (Sigma-Aldrich) by using a haematocyter. Proportions of the different leukocyte populations were determined by flow cytometry.

The following rat anti-mouse antibodies were used for analysis of cell composition in the pleural cavity: anti-CD19-APC (dilution 1∶25, BD Pharmingen, clone 1D3) as a marker of B cells; anti-CD5-PE (dilution 1∶25, BD Pharmingen, clone 53-7.3), a marker found on B1 cells and not on B2 cells; anti-CD3-PEcy5 (dilution 1∶25, BD Pharmingen, clone 17A2) as a marker of T cells; anti-F4/80-APC (dilution 1∶50; eBioscience, clone BM8), a marker of macrophages, SiglecF-PE (dilution 1∶40, BD Bioscience, clone E50-2440) as a marker of eosinophils, and Ly6G-PE-Cy7 (eBioscience, clone RB6-8C51∶40) as a marker of neutrophils. Controls were made with the appropriate isotype control. Acquisition and analyses were performed as described in Figure S1. Flow cytometry analysis was performed using a FACSCanto flow cytometer running the FACS DIVA software (BD Biosciences).

### PleCs culture and ELISA of culture supernatants, pleural wash fluids, and sera

PleCs were cultured in duplicate in 96-well plates, with 2.5×10^5^ cells/well in RPMI 1640 medium supplemented with 10% FCS, 2 mM L-glutamine (Sigma Aldrich), 100 U of penicillin per ml, and 100 µg of streptomycin per ml (Eurobio), and stimulated for 72 hours (h) with Ad filarial extract (10 µg/ml) or ConA (1 µg/ml) at 37°C in 5% CO2-enriched atmosphere.

Pleural wash fluids (dilution 1∶3), sera (dilution 1∶5) or culture cells supernatant (dilution 1∶3) collected from individual mice were assayed for cytokine content by enzyme-linked immunosorbent assay (ELISA) in duplicate. These assays were performed according to the manufacturer's recommendations, using CXCL12 ELISA kits (e-Bioscience or R&D) and IL-5 ELISA kit (e-Bioscience). Results are expressed as picograms by milliliter. Detection limits were 44 pg/ml for CXCL12 and 4 pg/ml for IL-5.

### Isolation, characterization of murine pleural mesothelial cells, CXCL12 titration and CXCR4 immunostaining

Mesothelial cells were obtained by a 20 minute trypsin digestion of visceral and parietal pleural mesothelium. Mice were injected with 1.2 mL of trypsin-EDTA 0.25% in the pleural cavity at 37°C. The external face of the pleural cavity was kept moist with PBS and massaged periodically to improve cell detachment. The cavity was then gently washed with 10 ml of RPMI supplemented with 10% of FCS to neutralize the trypsin.

The media containing the cells were centrifuged at 200 g for 5 minutes. The cells were washed three times in RPMI 1640 supplemented with 20% FCS, 2 mM L-glutamine, 100 U of penicillin per ml, 100 g of streptomycin per ml, gentamicine 250 µg/ml, 20 mM HEPES and then cultured in T25 flasks for 6–8 days at 37°C in a 5% CO2-enriched atmosphere until they were subconfluent. Mesothelial cells were characterized according to their shape and following cytokeratin 7 staining (1∶50, clone AE1/AE3, DAKO). Cells from 30 days p.i. infected-BALB/c and-C57BL/6 mice were seeded at 1 million cells per mL in 24-well plates and then stimulated or not with filarial extract (10 µg/ml). Supernatants were recovered after 48 hours of culture for CXCL12 titration by ELISA as described above.

After the first passage, mesothelial cells were grown on chamber slides for 2 days (Lab-Teck, Polylabo, France). Cells were treated with Brefeldin A (10 µg/ml) for the last 4 h of culture. Cells were washed and fixed with 4% paraformaldehyde in PBS for 10 min at 4°C, permeabilized with PBS 0.2% BSA, 0.05% Saponine buffer for 30 min at 4°C, incubated with the anti-CXCR4 polyclonal antibody (Thermo Scientific, France) for 30 min at 4°C, and finally incubated with secondary Ab goat anti-rabbit IgG-Alexa Fluor 488 (Molecular Probes). After 3 washes in PBS, slides were mounted with Fluoromount-G (Southern Biotechnology Associates). Images were taken using an inverted microscope Zeiss Axiovert 200 M piloted by the Zeiss Axiovision 4.4 software, acquired with a CCD camera Roper Scientific Coolsnap HQ, and analysed using the AxioVision LE program.

### Immunohistology of visceral pleural mesothelium

To expand the lungs of BALB/c and C57BL/6 mice and to preserve the structure of the visceral pleural mesothelium, they were injected through the trachea with 4% formaldehyde in cold PBS. The organs were then fixed for 48 h by immersion in 4% formaldehyde. Fixed samples were embedded in paraffin and 5-µm-thick sections were prepared. The tissues were deparaffinized with toluene and then hydrated using a series of decreasing concentrations of ethanol. The visceral pleural mesothelium was stained for cytokeratin 7, a marker of mesothelial cells (1∶50, clone AE1/AE3, DAKO) or CXCL12 (polyclonal antibody, 1∶500, eBioscience; and biotinylated monoclonal 1∶200, clone K15C, [69]). Binding of these antibodies was detected by HRP or HRP-linked universal secondary antibody (DAKO) and AEC substrate (DAKO). The sections were counterstained with a Mayer Hematoxylin solution. Four animals were studied for each condition and stained lung sections were examined by light microscopy. The level of CXCL12 was arbitrary scored as 0 (no detectable staining), 5 (intermediate staining), and 10 (high staining). Representative images were chosen for each staining intensity (negative, intermediate or high). The numerical scoring was confirmed by a second independent examination, blinded to the initial score.

### In vitro culture of larvae

L4-stage larvae were recovered 15 days after L3 inoculation by flushing the pleural cavity of jirds with cold PBS. The larvae were then washed twice by a medium constituted of RPMI 1640, 25% SVF, 2 mM L-glutamine (Sigma Aldrich), 100 U of penicillin per ml, 100 µg of streptomycin per ml (Eurobio), HEPES 20 mM, glucose 1.1%, vitamin C 75 µM, and BSA 3%.

L4 were seeded two per well in 24 well culture plates and the medium was changed each day from day 0 to day 5. Filariae were stimulated from day 1, with CXCL12 (1 or 10 nM), AMD3100 (25 µg/ml or 50 µg/ml), Chalcone C04 (1, 10 µM) or IL-5 (5 ng/ml). The general appearance of cultured worms was observed each day. Pictures were taken regularly for length measurement after immobilization of the worms by 30 minutes at 4°C.

### Statistics

The choice of statistical tests was based on sample size and on Bartlett's test when normal distributions of the errors were expected. Data from separate experiments were pooled when possible. Results were analyzed with two-way ANOVA in order to determine the effects of 2 factors, *i.e.* the strain and the treatment. Bonferroni's multiple comparisons post-test was used to compare treated groups to untreated groups in each mouse strain, or to compare the two strains similarly treated. For *in vitro* experiments, non-parametric Kruskall-Wallis's H-test was used. Pleural exudates cell composition was analyzed by MANOVA. Binomial generalized linear models (glm) were used to assess the effects of treatment and strain on growth delay. Multiple factorial analysis (MFA) was performed to represent the links between the variables involved in this study. Representation and data analysis were performed with R [70] or the GraphPad Prism 5 software. Statistically significant values are indicated as follows: *: p<0.05; **: p<0.01; and ***: p<0.001.

### Accession Numbers

The GenBank accession numbers used to search for the CXCR4 ortholog in *Brugia malayi* are HGNC:2561 for the human *CXCR4 gene* and MGI:109563for the murine CXCR4 gene.

## Supporting Information

Figure S1 **Differential kinetics of pleural chemokine levels between C57BL/6 and BALB/c mice.** Differential CCL2 (A) , CCL3 (B) and CCL11 (C) response between BALB/c mice (Bc) and C57BL/6 mice (B6) in pleural fluid during the course of infection. Pleural wash fluids (dilution 1∶3) were assayed for cytokine content by enzyme-linked immunosorbent assay (ELISA) in duplicate. These assays were performed according to the manufacturer's recommendations, using CCL2, CCL3 and CCL11 ELISA kits (Peprotech). Results are given in picograms per milliliter. Results are expressed as mean ± SEM of 3 pooled independent experiments each carried out with 6 mice per group. Two ways analysis of variance followed by Bonferroni multiple comparison test. *: p<0.05, **: p<0.005, ***: p<0.001.
(TIF)

Figure S2 **Cytometry analysis of pleural exudate cells.** A. Pleural exudate cells were characterized by FACS analysis. Cells were labelled with various antibodies and then analysed by flow cytometry (FACSCanto BD, FACS DIVA version 6.0). From left to right : gating of macrophages and eosinophils is defined on a F4/80/Siglec F plot: gate for macrophages is defined as high and intermediate expression of F4/80 combined to low to intermediate expression of Siglec F; gate for eosinophils is defined as high expression of Siglec F; gating of neutrophils is defined on high expression of Ly6G; gating of B and T cells is defined on a CD19/CD5 expression plot; gate for B cells is in the left top corner; gate for T cells is in the right bottom corner. B. Results presented are the mean ± SEM of 6 observations. MANOVA, significative effect of strain (on total number of PleCs), no effect of treatment.
(TIF)

Figure S3 **Differential production of chemokines by PleCS between C57BL/6 and BALB/c mice.** Differential CCL2 (A) , CCL3 (B) and CCL11 (C) responses between BALB/c mice (Bc) and C57BL/6 mice (B6) in PleC surpernatant. Pleural exudate cells (PleCs) were harvested by PBS washing 30 days post-filarial inoculation (F) or from naive mice (N). The cells were stimulated for 72 hours by a crude extract of adult (Ad) *L. sigmodontis* (10 µg/ml) or with 1 µg/ml of the mitogen Concanavalin A (C), or were left unstimulated (us). Levels of CCL2, CCL3, CCL11 were detected by ELISA (Peprotech) in the culture supernatant. Results are expressed as picograms by milliliter. Results are expressed as mean ± SEM of 3 pooled independent experiments each carried out with 6 mice per group. Two-way analysis of variance followed by Bonferroni multiple comparison test. *p<0.05, **p<0.005, ***p<0.001.
(TIF)

Figure S4 **Multiple factorial analysis of worm and immune parameters: focus on the first dimension.** A. Individuals plot in factorial plane (1, 2) shows a marked separation between the two strains. B. Correlation circle of the axis 1 and 2 presenting the patterns of responses in resistant versus susceptible mice: low number of filariae (nF), high recruitment of pleural exudates cells (PleCs), high CXCL12 and IL-5 concentration (CXCL12.LP, IL5.LP), high number of stage 4 larvae and fourth molting filariae (L4–M4).
(TIF)

Figure S5 **Schematic overview of the regulation of filarial survival and development by the CXCL12/CXCR4 axis in the pleural cavity.** A. The CXCL12/CXCR4 axis controls filarial survival in C57BL/6 non permissive mice. Survival of *L. sigmodontis* is represented in the pleural cavity of C57BL/6 and BALB/C mice, before and after treatments disrupting the CXCL12/CXCR4 axis. Without treatments (control), C57BL/6 mice pleural mesothelial cells produce high levels of CXCL12 that correlate with low levels of filariae and high numbers of pleural exudate cells. On the contrary, BALB/c mice pleural mesothelial cells produce low levels of CXCL12 that correlate with high levels of filariae and low numbers of pleural exudate cells. After CXCR4 blockade by AMD3100 treatment, mesothelial cells from C57BL/6 mice produce low levels of CXCL12; these mice also have a lower number of pleural exudate cells equivalent to the one of BALB/c mice and an intermediate number of filariae between C57BL/6 and BALB/c mice. After AMD3100 treatment in BALB/c mice, levels of CXCL12, numbers of pleural exudate cells and of filariae are in all points similar to untreated BALB/c mice. After chalcone C04 treatment, C57BL/6 mice have a low level of CXCL12, a high number of filariae and a low number of pleural exudate cells, all equivalent to BALB/c mice. After chalcone C04 treatment in BALB/c mice, levels of CXCL12, numbers of pleural exudate cells and of filariae are in all points similar to untreated BALB/c mice. B. *L. sigmodontis* development is dependant of the CXCL12/CXCR4 axis in both C557BL/6 and BALB/c mice. A hypothetical explanation of the effect of CXCL12 on the filarial development is presented. The mechanism relies on the existence of a *L. sigmodontis* CXCR4-like receptor (fCXCR4-like) and the capacity of chemokine receptors to be desensitized in presence of high levels of ligand. In C57BL/6 mice, the pleural cavity is rich in CXCL12. This high level could cause desensitization of the fCXCR4-like receptor, thus retarding the growth of the parasite. Inversely, BALB/c mice present a low level of CXCL12 in the pleural cavity that activates fCXCR4-like receptor thus favoring growth. After AMD3100 treatment, C57BL/6 and BALB/c mice present low level of CXCL12 in the pleural cavity. However, according to our *in vitro* data, AMD3100 could block a homolog/mimicry of CXCR4, thus retarding the growth. After chalcone C04 treatment, the CXCL12 level is also low in the pleural cavity of both C57BL/6 and BALB/c mice, but the interaction of the chalcone with CXCL12 could prevent CXCL12/fCXCR4-like interaction, thus retarding the growth of *L. sigmodontis*.
(TIF)
