# Supplementary figures and images for: The Chemokine CXCL12 Is Essential for the Clearance of the Filaria Litomosoides sigmodontis in Resistant Mice
Source: PLoS One. 2012 Apr 12;7(4):e34971. doi: 10.1371/journal.pone.0034971 (PMC3325259; doi:10.1371/journal.pone.0034971)

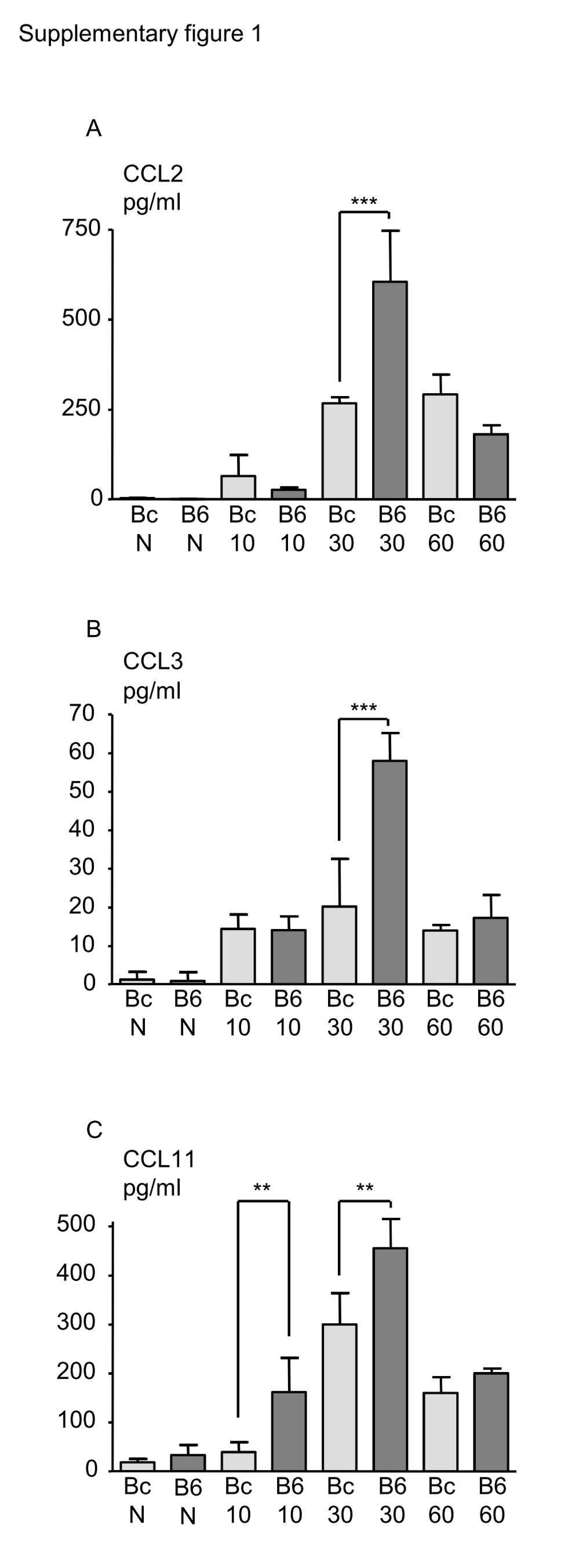

Supplement: Figure S1 — Differential kinetics of pleural chemokine levels between C57BL/6 and BALB/c mice. Differential CCL2 (A) , CCL3 (B) and CCL11 (C) response between BALB/c mice (Bc) and C57BL/6 mice (B6) in pleural fluid during the course of infection. Pleural wash fluids (dilution 1∶3) were assayed for cytokine content by enzyme-linked immunosorbent assay (ELISA) in duplicate. These assays were performed according to the manufacturer's recommendations, using CCL2, CCL3 and CCL11 ELISA kits (Peprotech). Results are given in picograms per milliliter. Results are expressed as mean ± SEM of 3 pooled independent experiments each carried out with 6 mice per group. Two ways analysis of variance followed by Bonferroni multiple comparison test. *: p<0.05, **: p<0.005, ***: p<0.001. (TIF) [file pone.0034971.s001.tif]

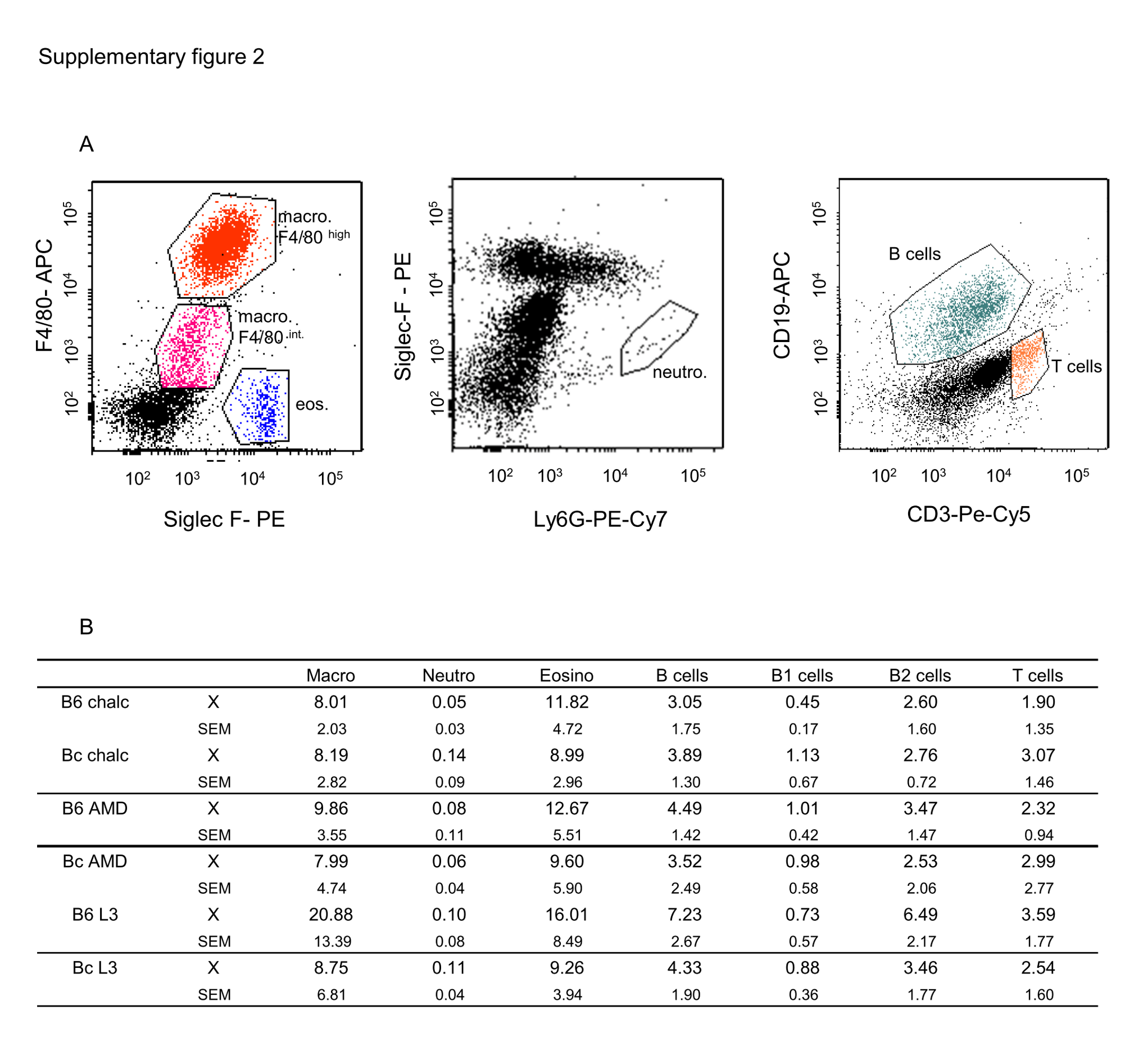

Supplement: Figure S2 — Cytometry analysis of pleural exudate cells. A. Pleural exudate cells were characterized by FACS analysis. Cells were labelled with various antibodies and then analysed by flow cytometry (FACSCanto BD, FACS DIVA version 6.0). From left to right : gating of macrophages and eosinophils is defined on a F4/80/Siglec F plot: gate for macrophages is defined as high and intermediate expression of F4/80 combined to low to intermediate expression of Siglec F; gate for eosinophils is defined as high expression of Siglec F; gating of neutrophils is defined on high expression of Ly6G; gating of B and T cells is defined on a CD19/CD5 expression plot; gate for B cells is in the left top corner; gate for T cells is in the right bottom corner. B. Results presented are the mean ± SEM of 6 observations. MANOVA, significative effect of strain (on total number of PleCs), no effect of treatment. (TIF) [file pone.0034971.s002.tif]

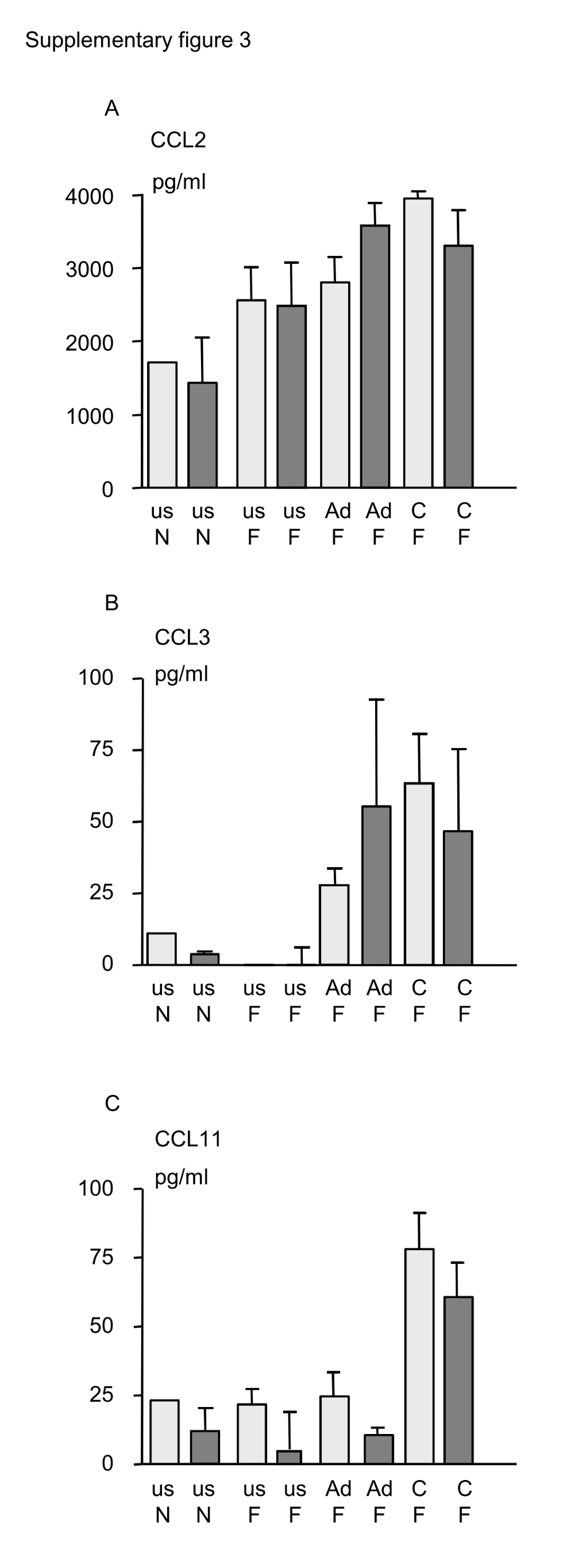

Supplement: Figure S3 — Differential production of chemokines by PleCS between C57BL/6 and BALB/c mice. Differential CCL2 (A) , CCL3 (B) and CCL11 (C) responses between BALB/c mice (Bc) and C57BL/6 mice (B6) in PleC surpernatant. Pleural exudate cells (PleCs) were harvested by PBS washing 30 days post-filarial inoculation (F) or from naive mice (N). The cells were stimulated for 72 hours by a crude extract of adult (Ad) L. sigmodontis (10 µg/ml) or with 1 µg/ml of the mitogen Concanavalin A (C), or were left unstimulated (us). Levels of CCL2, CCL3, CCL11 were detected by ELISA (Peprotech) in the culture supernatant. Results are expressed as picograms by milliliter. Results are expressed as mean ± SEM of 3 pooled independent experiments each carried out with 6 mice per group. Two-way analysis of variance followed by Bonferroni multiple comparison test. *p<0.05, **p<0.005, ***p<0.001. (TIF) [file pone.0034971.s003.tif]

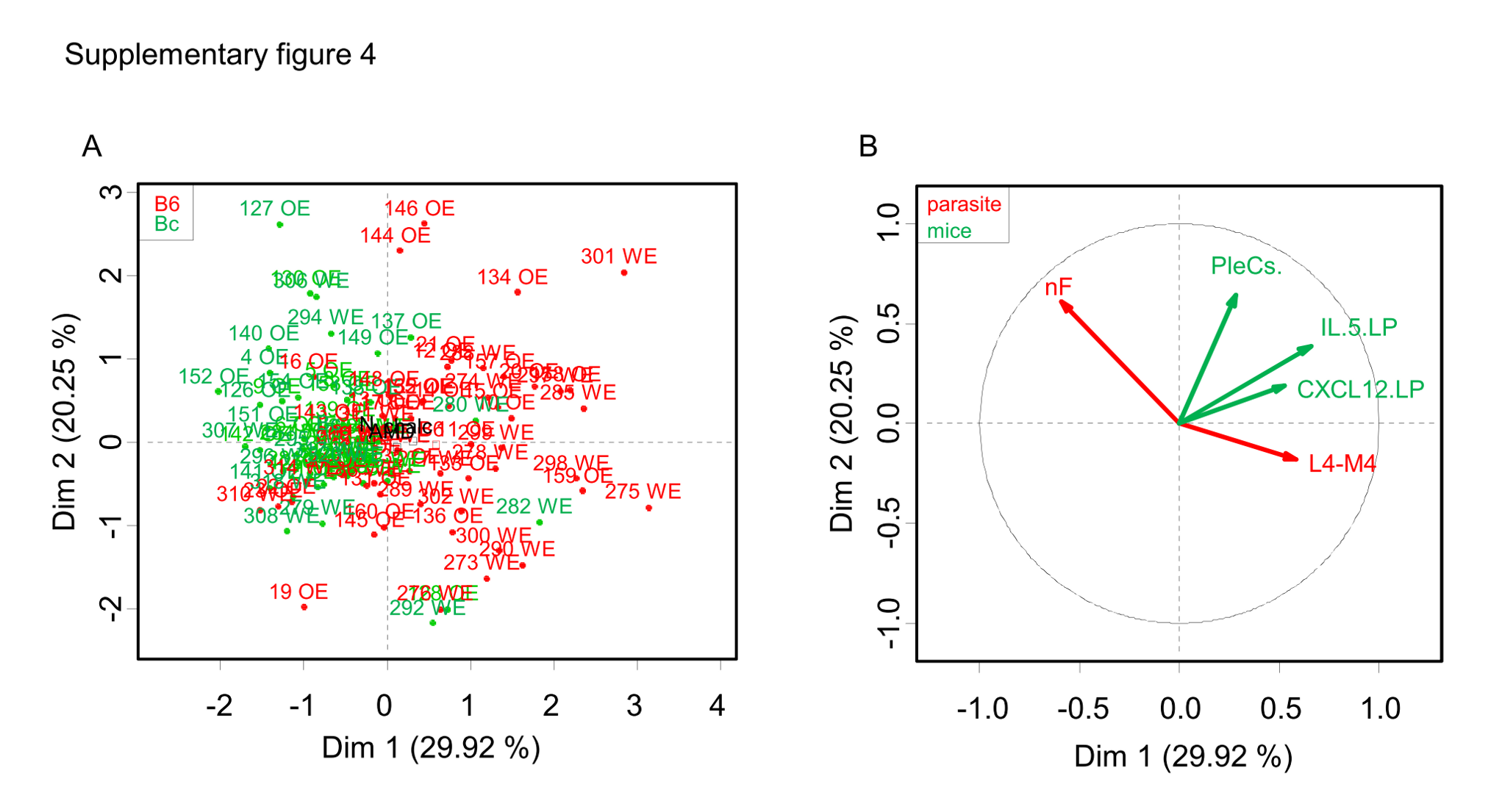

Supplement: Figure S4 — Multiple factorial analysis of worm and immune parameters: focus on the first dimension. A. Individuals plot in factorial plane (1, 2) shows a marked separation between the two strains. B. Correlation circle of the axis 1 and 2 presenting the patterns of responses in resistant versus susceptible mice: low number of filariae (nF), high recruitment of pleural exudates cells (PleCs), high CXCL12 and IL-5 concentration (CXCL12.LP, IL5.LP), high number of stage 4 larvae and fourth molting filariae (L4–M4). (TIF) [file pone.0034971.s004.tif]

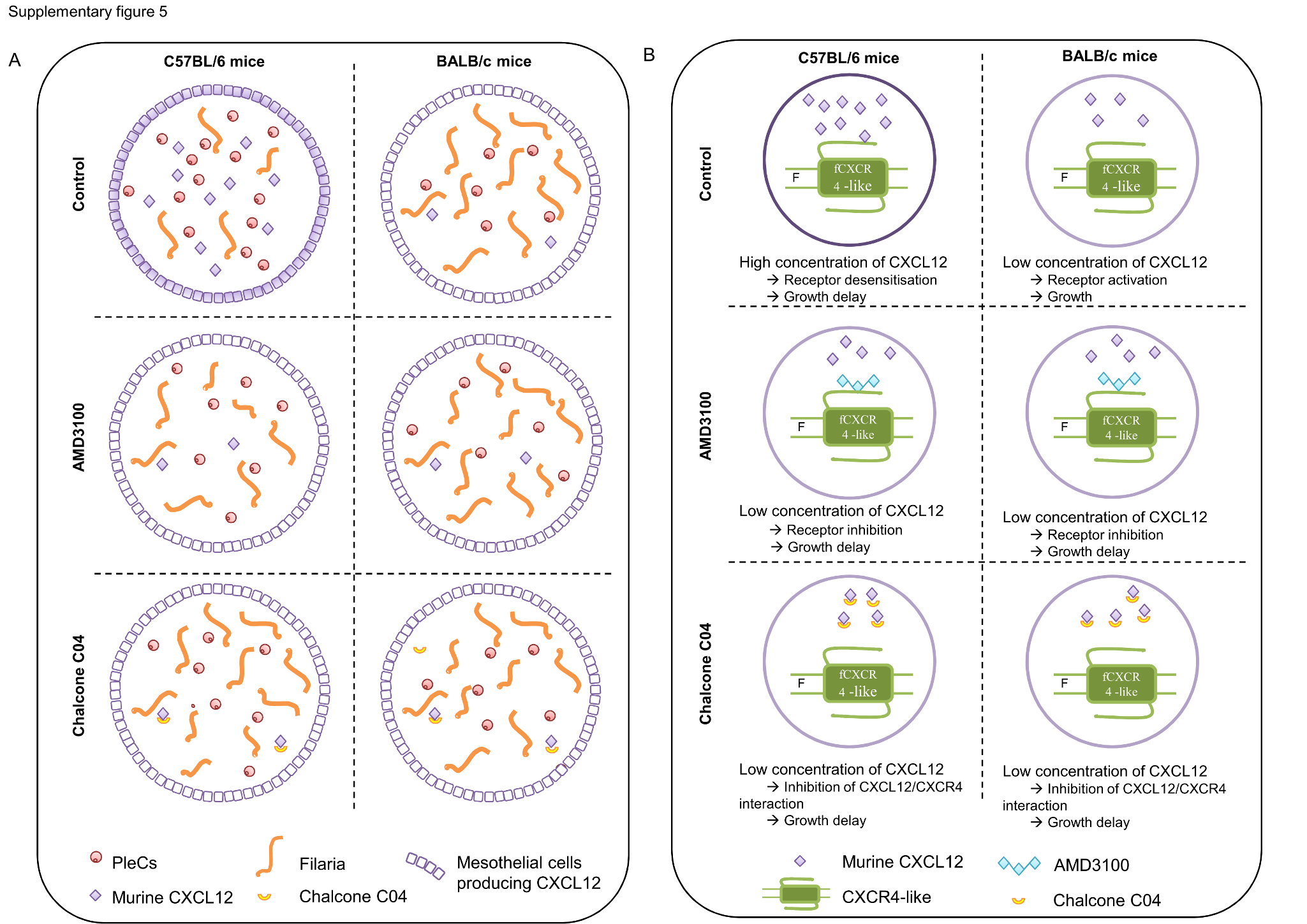

Supplement: Figure S5 — Schematic overview of the regulation of filarial survival and development by the CXCL12/CXCR4 axis in the pleural cavity. A. The CXCL12/CXCR4 axis controls filarial survival in C57BL/6 non permissive mice. Survival of L. sigmodontis is represented in the pleural cavity of C57BL/6 and BALB/C mice, before and after treatments disrupting the CXCL12/CXCR4 axis. Without treatments (control), C57BL/6 mice pleural mesothelial cells produce high levels of CXCL12 that correlate with low levels of filariae and high numbers of pleural exudate cells. On the contrary, BALB/c mice pleural mesothelial cells produce low levels of CXCL12 that correlate with high levels of filariae and low numbers of pleural exudate cells. After CXCR4 blockade by AMD3100 treatment, mesothelial cells from C57BL/6 mice produce low levels of CXCL12; these mice also have a lower number of pleural exudate cells equivalent to the one of BALB/c mice and an intermediate number of filariae between C57BL/6 and BALB/c mice. After AMD3100 treatment in BALB/c mice, levels of CXCL12, numbers of pleural exudate cells and of filariae are in all points similar to untreated BALB/c mice. After chalcone C04 treatment, C57BL/6 mice have a low level of CXCL12, a high number of filariae and a low number of pleural exudate cells, all equivalent to BALB/c mice. After chalcone C04 treatment in BALB/c mice, levels of CXCL12, numbers of pleural exudate cells and of filariae are in all points similar to untreated BALB/c mice. B. L. sigmodontis development is dependant of the CXCL12/CXCR4 axis in both C557BL/6 and BALB/c mice. A hypothetical explanation of the effect of CXCL12 on the filarial development is presented. The mechanism relies on the existence of a L. sigmodontis CXCR4-like receptor (fCXCR4-like) and the capacity of chemokine receptors to be desensitized in presence of high levels of ligand. In C57BL/6 mice, the pleural cavity is rich in CXCL12. This high level could cause desensitization of the fCXCR4-like receptor, [file pone.0034971.s005.tif]
